# Supplementary material for: Aggressive behaviour of psychiatric patients with mild and borderline intellectual disabilities in general mental health care
Source: PLoS One. 2022 Oct 3;17(10):e0272502. doi: 10.1371/journal.pone.0272502 (PMC9529125; doi:10.1371/journal.pone.0272502)
Supplement: S1 File — (DOCX) [file pone.0272502.s003.docx]

Explanation of Methods

As stated on the website of Plos one, from March 2022, published articles that link to research data in the OSF, Figshare or Dryad repositories will display an “Accessible Data” feature, as part of a project intended to increase sharing and reuse of data in repositories. The data of the current study may be found at the OSF repository in a project entitled: Aggressive behaviour of psychiatric patients with Mild and Borderline Intellectual Disabilities in general Mental Health Care. This document and an data file translated in English within Excel format were uploaded. Below we provide the methods section and after each section a brief explanation of the neccesary syntaxes.

At the level of the patient, we identified whether a patient had shown an aggressive incident and whether a patient had shown outwardly directed physical aggression incidents against persons (so not against themselves). The total number of SOAS-R incidents per patient reported between 2014 and 2019 was also counted. Differences in the number of incidents between patients with or without MID/ BIF were tested using the Kruskal-Wallis rank order test because of extremely skewed frequencies. As mentioned earlier, the SCIL outcomes were categorised in scores of 19 and less, representing assumed MID/BIF and scores of 15 and less, representing assumed MID. BIF, MID and patient characteristics were cross-tabulated with having shown aggression incidents and physical aggression incidents against persons. We calculated chi-square statistics and Odds ratios to investigate the significance of the differences and the increased risk of showing (physical) aggression in relation to patient characteristics. Below, we show the general syntaxis. Next to these we also performed the same analyses on age categories, gender, and diagnostic categories.

CROSSTABS CROSSTABS

/TABLES=Indic_BIF scil15 BY aggression /TABLES=Indic_BIF scil15 BY agression
 /FORMAT=AVALUE TABLES BY wardtype

/STATISTICS=CHISQ RISK /FORMAT=AVALUE TABLES

/CELLS=COUNT COLUMN /STATISTICS=CHISQ RISK

/COUNT ROUND CELL. /CELLS=COUNT COLUMN

/COUNT ROUND CELL.

We also performed a logistic regression analysis to understand the association of these variables with having shown any aggression or physical aggression corrected for one another. A forward entry and backward deselection procedure were used. All variables selected from the EMC were entered in the analysis. Thus gender, age categories, diagnosis, MID or BIF as assessed with the SCIL. For the forward selection, variables with associations having a p-value of <0.2 were included in the logistic regression analysis, following the relevance criterion proposed by Hosmer and Lemeshow [28].

These were entered in 3 blocks: the demographic variables, the diagnoses, and the response categories in the SCIL. Below, we present the general syntaxis of the logistic regression

LOGISTIC REGRESSION VARIABLES aggressive

/METHOD=BSTEP(WALD) Gender age_cat scil_pos16_19 scil_pos15_less anxiety depression

bipolar schizophrenia Developmental_disorder Drug_abuse_disorder lowgaf

/CONTRAST (Gender)=Indicator(1)

/CONTRAST (age_cat_1)=Indicator(1)

/CONTRAST (age_cat_2)=Indicator(1)

/CONTRAST (scil_pos16_19)=Indicator(1)

/CONTRAST (scil_pos15_less)=Indicator(1)

/CONTRAST (anxiety)=Indicator(1)

/CONTRAST (depression)=Indicator(1)

/CONTRAST (bipolar)=Indicator(1)

/CONTRAST (schizophrenia)=Indicator(1)

/CONTRAST (Developmental_disorder)=Indicator(1)

/CONTRAST (Drug_abuse_disorder)=Indicator(1)

/CONTRAST (lowgaf)=Indicator(1)

/PRINT=GOODFIT CI(95)

/CRITERIA=PIN(0.05) POUT(0.10) ITERATE(20) CUT(0.5).

Next, Poisson regression was applied to the number of incidents as we may expect a skewed distribution, and the number of incidents represents a count. Before applying the regression, the distribution of the number of incidents was tested. We applied forward entry and backward deselection to investigate which patient characteristics predicted the number of aggression incidents. We present the β, which as a rate ratio can be interpreted as a growth or downturn rate [29]. Below, we present the general syntaxis of the GLM module in Spss, which we used for the poisson regression on the number of aggression incidents

* Generalized Linear Models.

GENLIN Number_incidents_total BY gender age_cat anxiety depression bipolar schizophrenia Developmental_disorder Drug_abuse_disorder lowgaf (ORDER=DESCENDING)

/MODEL gender age_cat anxiety depression bipolar schizophrenia Developmental_disorder Drug_abuse_disorder lowgaf INTERCEPT=YES

DISTRIBUTION=POISSON(1)

/CRITERIA METHOD=FISHER(1) SCALE=1 COVB=MODEL MAXITERATIONS=100 MAXSTEPHALVING=5

PCONVERGE=1E-006(ABSOLUTE) SINGULAR=1E-012 ANALYSISTYPE=3(WALD) CILEVEL=95 CITYPE=WALD

LIKELIHOOD=FULL

/MISSING CLASSMISSING=EXCLUDE

/PRINT CPS DESCRIPTIVES MODELINFO FIT SUMMARY SOLUTION (EXPONENTIATED).

In the case when authors want to repeat these analyses, bur need support, please contact the last author of the publication: [e.noorthoorn@ggnet.nl](mailto:e.noorthoorn@ggnet.nl)
