# Supplementary material for: Aggressive behaviour of psychiatric patients with mild and borderline intellectual disabilities in general mental health care
Source: PLoS One. 2022 Oct 3;17(10):e0272502. doi: 10.1371/journal.pone.0272502 (PMC9529125; doi:10.1371/journal.pone.0272502)
Supplement: Attachment — Submitted filename: 15-06-2022 PLOS ONE ID agression rebuttal 2_.docx [file pone.0272502.s005.docx]

PONE-D-21-40785R1

Aggressive behaviour of psychiatric patients with Mild and Borderline Intellectual Disabilities in general Mental Health Care

PLOS ONE

1. If the authors have adequately addressed your comments raised in a previous round of review and you feel that this manuscript is now acceptable for publication, you may indicate that here to bypass the "Comments to the Author" section, enter your conflict of interest statement in the "Confidential to Editor" section, and submit your "Accept" recommendation.

Reviewer #1: (No Response)

________________________________________

2. Is the manuscript technically sound, and do the data support the conclusions?

The manuscript must describe a technically sound piece of scientific research with data that supports the conclusions. Experiments must have been conducted rigorously, with appropriate controls, replication, and sample sizes. The conclusions must be drawn appropriately based on the data presented.

Reviewer #1: Yes

________________________________________

3. Has the statistical analysis been performed appropriately and rigorously?

Reviewer #1: Yes

________________________________________

4. Have the authors made all data underlying the findings in their manuscript fully available?

The PLOS Data policy requires authors to make all data underlying the findings described in their manuscript fully available without restriction, with rare exception (please refer to the Data Availability Statement in the manuscript PDF file). The data should be provided as part of the manuscript or its supporting information, or deposited to a public repository. For example, in addition to summary statistics, the data points behind means, medians and variance measures should be available. If there are restrictions on publicly sharing data—e.g. participant privacy or use of data from a third party—those must be specified.

Reviewer #1: Yes

________________________________________

5. Is the manuscript presented in an intelligible fashion and written in standard English?

PLOS ONE does not copyedit accepted manuscripts, so the language in submitted articles must be clear, correct, and unambiguous. Any typographical or grammatical errors should be corrected at revision, so please note any specific errors here.

Reviewer #1: No

________________________________________

6. Review Comments to the Author

Please use the space provided to explain your answers to the questions above. You may also include additional comments for the author, including concerns about dual publication, research ethics, or publication ethics. (Please upload your review as an attachment if it exceeds 20,000 characters)

Reviewer #1: Thank you for the opportunity to re-review this manuscript. The authors have addressed most of my concerns. Remaining issues are listed below – I have also commented on some new sections of text.

Response #1We thank the reviewer for this comment. Also for the new comments. We think the article improved again a lot.

#1. Again I would encourage use of person-first language, e.g.

a) changing "MID/BIF patients" (pg 2, line 67) to "people with MID/BIF"; "

b) SMI patients" (page 5, line 137, line 142, pg 6 line 174) to "people with SMI";

c) "the homeless" (page 6, line 168) to "people experiencing homelessness";

d) "MID or BIF cases" (page 8, line 253) to "people with MID or BIF";

e) "MID patients" (page 8, line 257) to "people with MID"

#1. response: We have adopted the recommendations. The only difference is that this study was conducted in the mental health care, where we speak of patients. Therefore, we have used patients instead of people throughout the draft.

2. Page 2, line 87, suggest changing "the more severely intellectually impaired patients" to "people with more severe intellectual disability"

Response 2: we have changed this in ‘patients with more severe intellectual disability’.

3. Page 5, line 135, "Serious Mentally Ill patients' should read "people with serious mental illness (SMI)".

Response 3: Again we changed this, but in patients with serious mental illness

4. Again, terms such as mild intellectual disability (pg 2, line 63), borderline intellectual functioning (pg 2, line 63), mental health care (pg 2, line 64; page 4, line 120), challenging behaviour (pg 2, line 73) do not need to have the first letter of each word capitalised.

Response 4: Thank you for being so precise. We changed this all.

5. Page 11, line 336: intellectual disability should be abbreviated to ID in the following sentence- "As we can learn from ID studies based on interviews with people with intellectual disability [10, 37], people with ID often experience.."

Response 5: Again thank you for being so precise. We changed this.

6. Page 9, line 271: should "Patients with bipolar disorder… were associated with an increased risk of" read "Diagnoses of bipolar disorder… were associated with an increased risk of"?

Response 6: We changed this.

7. Page 9, line 276-77: check for typographical errors – e.g. "associated to" should read "associated with"

Response 7: Most were "associated with". With search and change we detected only one "associated to "in the whole article, and corrected this.

8. Page 9, line 280-285: inconsistent use of "patients who screened positive for BIF or MID were associated with . . . and "MID was associated with". It would be helpful for the reader to be consistent. I would also recommend this section be reviewed by a statistician or biostatistician to ensure the results are appropriately differentiated in text from those of the Poisson regression. E.g. if logistic regression would be determining risk for having at least one aggressive incident, while the Poisson would look at the number of incidents.

Response 8:

An experienced statistician was one of the authors (Noorthoorn) and carefully reviewed this section. We substantially reformulated the complete section, using for the logistic regression ‘being at risk’ in stead of the more vague ‘associated with’. With respect to the poisson regression we presented the beta (β).

Logistic regression

The logistic regression analysis showed that patients who screened positive for BIF (OR=2.00, p=0.003) or MID (OR 2.89, p<0.001) were more at risk to show aggressive incidents, as well as the patients with the diagnoses bipolar disorder (OR 3.07, p<0.001), schizophrenia (OR 2.75, p<0.001), and a low GAF (OR 1.72, p=0.005). Logistic regression analysis with physical aggression as outcome showed that patients with MID (OR 2.50, p<0.001), a bipolar disorder (OR 3.13, p=0.007) or schizophrenia (OR 4.04, p<0.001) were more at risk to show aggressive incidents.

Poisson regression

These findings were underlined by the poisson regression of the number of physical aggression incidents per patient. This showed anxiety disorder (β=0.62, p<0.001), bipolar disorder (β=1.63, p<0.001), schizophrenia (β=1.12, p<0.001), developmental disorder (β)=0.69, p<0.001), drug abuse disorder (β=1.18, p<0.001) and a SCIL below 15 (β=0.61, p<0.001) were all related to more incidents.

9. Page 11, line 339: if using the term challenging behaviour, then this should be used here as well – behaviours that challenge is an alternative expression preferred by some.

Response 9: We changed this to challenging behaviour.

10. Page 11, lines 356-360- The authors may wish to include some citations for statements included here.

Response 11:

Thank you for offering some references. We included a reference to The NICE guidelines and to the first two references (Whittle et al and Mesa et al. We did not citate because, in our opinion, this is beyond the scope of this article.

E.g. for recommendations regarding the prevention and management of behaviours that challenge: The NICE guidelines here https://www.nice.org.uk/guidance/ng11/resources/challenging-behaviour-and-learning-disabilities-prevention-and-interventions-for-people-with-learning-disabilities-whose-behaviour-challenges-1837266392005.

Regarding mental health services frequently being ill-equipped to meet the needs of people with intellectual disability:

Whittle, E.L.; Fisher, K.R.; Reppermund, S.; Lenroot, R.; Trollor, J. Barriers and Enablers to Accessing Mental Health Services for People With Intellectual Disability: A Scoping Review. J. Ment. Health Res. Intellect. Disabil. 2018, 11, 69–102.

Mesa, S.; Tsakanikos, E. Attitudes and self-efficacy towards adults with mild intellectual disability among staff in acute psychiatric wards: An empirical investigation. Adv. Ment. Health Intellect. Disabil. 2014, 8, 79–90.

We did not include the next publications suggested:

Evans, E.; Howlett, S.; Kremser, T.; Simpson, J.; Kayess, R.; Trollor, J. Service development for intellectual disability mental health: A human rights approach. J. Intellect. Disabil. Res. 2012, 56, 1098–1109.

Weiss, J.A.; Lunsky, Y.; Gracey, C.; Canrinus, M.; Morris, S. Emergency Psychiatric Services for Individuals with Intellectual Disabilities: Caregivers' Perspectives. J. Appl. Res. Intellect. Disabil. 2009, 22, 354–362.

Donner, B.; Mutter, R.; Scior, K. Mainstream In-Patient Mental Health Care for People with Intellectual Disabilities: Service User, Carer and Provider Experiences. J. Appl. Res. Intellect. Disabil. 2010, 23, 214–225.

11. Page 11, line 363, the I in ID has been deleted in track changes but should be retained

Response 11: Thank you for your attention. We changed this.

12. Page 11, like 367- can the authors include a reference to support the link between staff attitudes toward people with intellectual disability and the prevention and detection of aggressive incidents.

Response 12: We included the next references to support this link:

van den Bogaard KJHM, Nijman HLI, Embregts PJCM. Attributional styles of support staff working with people with intellectual disabilities exhibiting challenging behaviour. J Appl Res Intellect Disabil. 2020 May;33(3):465-474.

Knotter MH, Wissink IB, Moonen XM, Stams GJ, Jansen GJ. Staff's attitudes and reactions towards aggressive behaviour of clients with intellectual disabilities: a multi-level study. Res Dev Disabil. 2013 May;34(5):1397-407.

13. Page 12, line 370-372, it could be made clearer here that the authors are recommending further work specifically focusing on aggressive behaviour among people with intellectual disability within the mental health service setting, including how this compares to the broader population of people with intellectual disability (if that is what they are recommending).

Response 13:

Thank you for your comment. The lines 370-372 concern not further work (etc.) but concerns about giving staff and nurses a better opportunity for personal tailormade treatment and attitude toward patients with ID. We explained in lines 376-381 more precisely what kind of future research is recommended.

We changed the sentence and added an extra sentence ( and reference) to be more clear;

" In mental health care, future studies should examine the causes and reasons for aggression incidents in patients with MID/BIF and learn more about how these patients differ or resemble those of people in ID studies. For example, we do not yet know much about the mental problems in general in the patient group with ID in mental health care and how this affects behaviour, including how this compares to the broader population of people with intellectual disability and mental health problems [..].

14. Page 12- line 372- in the sentence starting "for example, we do not yet know much about…" it is not clear whether the authors are referring to mental health diagnosis profiles of their cohort specifically or mental health service users with intellectual disability more generally. This should be made more clear. Also, MHC should not be abbreviated here as in previous mentions it is expanded as mental health care.

Response 14:

As explained in our response 13, we tried to be more clear.

MCH has been changed in mental health care.

15. Page 12, line 377, suggest including references to supporting statements about the usefulness of positive behaviour

support.

Response 15: we added the next reference: Lewis N, Reynolds E, Vale M, Keenan E, Hartland A, Haines M, Davies BE. An evaluation of positive behavioural support implemented within an intensive community support service for people with learning disabilities who present with behaviours that challenge. J Intellect Disabil. 2021 Sep;25(3):348-356.

________________________________________

7. PLOS authors have the option to publish the peer review history of their article (what does this mean?). If published, this will include your full peer review and any attached files.

If you choose "no", your identity will remain anonymous but your review may still be made public.

Do you want your identity to be public for this peer review? For information about this choice, including consent withdrawal, please see our Privacy Policy.

Reviewer #1: No
